# Effects of different full squat training volumes matched for fatigue on strength gains, neuromuscular adaptations, and muscle hypertrophy

**DOI:** 10.5114/biolsport.2026.157999

**Published:** 2026-03-09

**Authors:** José Antonio Páez-Maldonado, Clara Cano-Castillo, Pedro Jesús Cornejo-Daza, Juan Sánchez-Valdepeñas, Luis Rodiles-Guerrero, Mathias Wernbom, Manuel Ortega-Becerra, Fernando Pareja-Blanco

**Affiliations:** 1 University of Osuna (Centre attached to the University of Seville), Osuna, Spain; 2 Science-Based Training Research Group, Physical Performance and Sports Research Center (CIRFD), Universidad Pablo de Olavide, Seville, Spain; 3 Faculty of Sports Sciences, Department of Sports and Computer Sciences, Universidad Pablo de Olavide, Seville, Spain; 4 Department of Human Movement and Sport Performance, University of Seville, Seville, Spain; 5 Physical Education and Sports Department, Cardenal Spínola CEU Andalucía University, Bormujos, Sevilla, Spain; 6 Department of Health and Rehabilitation, Institute of Neuroscience and Physiology, Sahlgrenska Academy, University of Gothenburg, Gothenburg, Sweden

**Keywords:** Cross-sectional area, Dose-response, Electromyography, Fatigue management, Velocity-based training, Velocity loss

## Abstract

To investigate the effects of three full squat (SQ) training volumes, matched for fatigue, on jump performance, muscle strength, neuromuscular adaptations, and muscle hypertrophy. Thirty-six resistance-trained men were randomized into three groups: low (LOW), moderate (MOD), and high (HIG) volume. All groups trained SQ twice a week, with relative intensities increasing from 70% to 85% 1RM over the 8-week training period. The total volume accumulated was 48, 144, and 312 repetitions for LOW, MOD, and HIG, respectively, during the 16 training sessions. To isolate the effect of training volume by minimizing fatigue accumulation across repetitions, short rest periods were inserted between repetitions. The following tests were performed: 1) cross-sectional area (CSA) of vastus lateralis; 2) countermovement jump (CMJ); 3) maximal isometric SQ contraction; 4) progressive loading SQ test; and 5) fatigue SQ test. MOD achieved the greatest gains in the progressive loading SQ test (“group-by-time” interaction: p = 0.02, effect sizes (ES): 0.87, 2.52, and 1.12, for LOW, MOD, and HIG, respectively). HIG showed the greatest increases in the electromyography amplitude during this test (“group-by-time” interaction: p = 0.03, ES: -0.13, 0.52, and 0.88, for LOW, MOD, and HIG). All groups showed significant increases in CSA, without significant differences between them (“group-by-time” interaction: p = 0.34, ES: 0.52, 0.41, and 0.56, for LOW, MOD, and HIG). Short inter-repetition rest periods enabled all training volumes to induce significant hypertrophy, while moderate volumes optimized strength gains and high volumes maximized neuromuscular activation, highlighting volume-specific adaptations in SQ training.

## INTRODUCTION

The level of effort, defined as the number of repetitions performed relative to the maximum possible that can be completed within the set against a given load [1], plays a key role in resistance training (RT) by shaping both the acute responses and long-term adaptations [1–4]. Higher levels of effort lead to a gradual decrease in repetition velocity [1–5]. This decline serves as a valuable indicator for quantifying fatigue in real-time, as research has shown that fatigue levels systematically rise as the level of effort within the set increases [1–4]. Specifically, RT programs with the same intensity but different magnitudes of velocity loss (VL) thresholds within a set lead to unique adaptations; for instance, a high VL (~40%) in the full-squat (SQ) exercise induces greater hypertrophy than lower VL levels (10–20%) [3]. However, excessive VL may shift muscle fibers toward slower phenotypes, potentially impairing performance in high-velocity actions [6].

Previous research has shown an inverted U-shaped relationship between performance gains and the level of effort within the set, objectively assessed through VL [3, 7]. Pareja-Blanco et al. [3] analyzed the effect of four VL thresholds (0%, 10%, 20%, and 40%) over 8 weeks on the SQ exercise, using the same relative load [from 70% to 85% of one-repetition maximum (1RM)]. The RT program with 10–20% VL magnitudes produced greater strength and jump performance improvements than training with 40% VL. This suggests the existence of an optimal level of fatigue, beyond which no further performance improvements occur, and adaptations may be lower than those observed with lower effort levels [3, 7]. However, the mechanisms underlying these findings remain unclear, as higher VL thresholds are associated with increased fatigue and greater volume.

Therefore, it is uncertain whether the primary factor driving these training-induced adaptations is: (a) fatigue, (b) volume, or (c) the interaction between both. While numerous studies have examined the effects of different training volumes [8, 9, 10], none have controlled for the potential influence of fatigue levels associated with a given volume. To attribute training effects specifically to volume rather than fatigue accumulated within a set, it is essential to design studies that compare different training volumes while maintaining similar fatigue levels. One way to control this factor is by monitoring lifting velocity in each repetition. To mitigate the increase in VL associated with higher volumes, brief rest periods within a set (i.e., inter-repetition rests) could be introduced [11, 12]. Additionally, these recovery periods could be individualized based on performance in each repetition, aiming to sustain performance (i.e., maintaining lifting velocity) as effectively as possible. We previously investigated the effects of different training volumes in the bench-press exercise, in which fatigue was controlled by incorporating inter-repetition rest periods, on strength gains and neuromuscular adaptations [13]. We found an inverted U-shaped relationship between training volume and performance gains, suggesting that moderate volumes maximize strength gains. The SQ is a multi-joint movement that engages greater muscle mass, involves higher levels of neuromuscular activation, and imposes different mechanical and metabolic demands compared to the bench press [1]. Given these distinctions, the effect of training volume *per se* on strength gains and neuromuscular adaptations remains to be assessed in a lower-body context. Therefore, the aim of this study was to investigate the effects of three SQ-based RT programs with varying volumes but controlled by fatigue through the use of short inter-repetition rest periods on strength performance, neuromuscular adaptations, and muscle hypertrophy.

Based on the inverted U-shaped relationship between training volume and performance gains, it was hypothesized that a moderate training volume would produce strength improvements similar to, or greater than, those achieved with high or low volumes. Finally, we hypothesized that training volumes would not elicit significant differences in muscle hypertrophy.

## MATERIALS AND METHODS

### Subjects

The sample size was calculated using G*Power (Version 3.1.9.2, Heinrich-Heine-Universität Düsseldorf, Düsseldorf, Germany) with the following parameters: statistical test: repeated measures ANOVA, within-between interaction; effect size (ES) = 0.4, based on ES observed for 1RM in previous literature examining different VL in SQ [3]; and α error probability (0.05) and power (0.95), three groups and two measurements, which resulted in a sample size of 10 subjects per group.

Considering potential dropouts, 45 (15 subjects per group) moderately strength-trained men (25.1 ± 5.1 years; 1.78 ± 0.06 m; = 76.9 ± 10.7 kg; 1RM relative to body mass = 1.35 ± 0.22) volunteered to participate in this study. The subjects were randomly assigned to one of three groups, following an ABCCBA design based on their 1RM. Participants had at least one year of RT experience and the ability to perform the SQ exercise with proper technique. Exclusion criteria included musculoskeletal injuries, use of performanceenhancing substances, and participation in other RT. Nine participants dropped out for reasons external to the investigation, leaving a final sample of 36 subjects (LOW: n = 12, MOD: n = 12, HIG: n = 12). Participants were instructed to refrain from engaging in any additional physical activity outside of the prescribed training program. Participants were informed about the purpose, testing procedures, and potential risk of injury before signing their informed consent. The study was approved by the ethics committee (Reference: 1547-N-19), following the Declaration of Helsinki.

### Study design

**FIG. 1:**
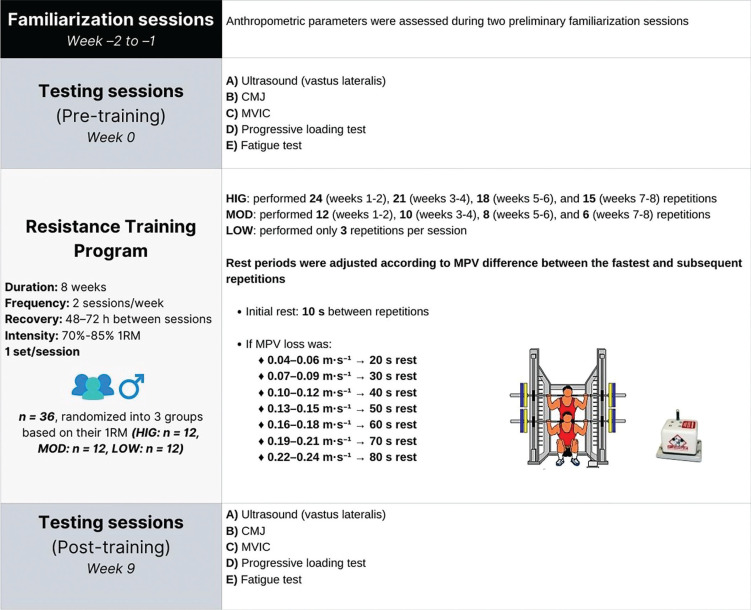
Schematic representation of the study design before and after 8 weeks of a resistance training program. CMJ: countermovement jump; MVIC: maximal voluntary isometric contraction; MPV: mean propulsive velocity; 1RM: one-repetition maximum; HIG: highvolume training group; MOD: moderate-volume training group; LOW: low-volume training group.

A longitudinal research design was used to compare the effects of three SQ training programs at 70–85% 1RM over 8 weeks (two times per week) with different training volumes (LOW: 48 repetitions, MOD: 144 repetitions, HIG: 312 repetitions). These volumes were selected to replicate those used in a previous study that demonstrated an inverted U-shaped relationship between strength gains and VL thresholds in the SQ exercise at intensities from 70% to 85% 1RM [3]. Specifically, the 0%, 20%, and 40% VL groups completed 48, 144, and 312 total repetitions, respectively. However, as previously noted, the observed training effects were likely the result of an interaction between fatigue and volume. To address this, a short inter-repetition rest period was added to isolate the impact of training volume, which is trying to minimize fatigue accumulation throughout repetitions. The rest duration was individualized based on the performance impairment induced in each repetition. Please refer to the Training Program section for details. Participants were instructed not to engage in other physical activities that could affect their performance. The researchers supervised all training sessions in a research laboratory under the same environmental conditions (20°C and 60% humidity) and at the same time of day (± 1 hour). The three groups were assessed twice: 48 hours before the start of the RT program (Pretraining) and 72 hours after the last training session (post-training). All subjects completed the intervention. Testing sessions were a) ultrasound measurements of the vastus lateralis muscle, b) countermovement jump (CMJ), c) maximal voluntary isometric contraction (MVIC) in the SQ exercise, d) progressive loading test in SQ, and e) fatigue SQ test. Electromyography (EMG) was measured in all SQ tests (Figure 1). Subjects received velocity feedback and verbal cues from researchers after every repetition during testing and training sessions. Anthropometric parameters (height and body mass) were assessed during two preliminary familiarization sessions.

### Testing Procedures

#### Ultrasonography

The cross-sectional area (CSA) of the vastus lateralis muscle was assessed through B-mode ultrasonography (MyLab 25, EsaoteBiomedica, Italy) with a 50 mm linear probe and 5–12 MHz. Participants were instructed to lie supine with their knees slightly flexed to 150° (180° represents full knee extension) on a foam roll to capture ultrasound images. After 15 minutes in the described position, the length of the vastus lateralis muscle was measured, defined as the distance between the edge of the lateral condyle of the femur and the top of the greater trochanter of the right leg [14]. An extended field-of-view mode was used to record the CSA of the vastus lateralis. To ensure the straight path of the probe, adhesive tapes were placed on the participants’ skin, and ultrasound gel was applied to the proximal and distal boundaries of the transverse path. A panoramic image was taken from a medial to lateral perspective, carefully and at a constant speed, passing along the established path, keeping the probe perpendicular to the skin surface, and applying minimal pressure to avoid compressing the tissues being evaluated. Three images were recorded at the following vastus lateralis lengths: 40%, 57.5%, and 75%, obtaining CSA40, CSA57.5, and CSA75, respectively. The same researcher analyzed these images digitally (ImageJ 1.51j8, NIH), encircling the boundaries (aponeuroses) of the vastus lateralis muscle. Each variable was analyzed based on two images. The third image was evaluated if a coefficient of variation (CV) greater than 5% was detected. For further analysis, the average value of all assessed images was taken. Consistency in measurement sites pre-post training was achieved by recording probe positions onto a transparent acetate and connective tissues as landmarks. Muscle volume (cm^3^) was estimated using the method based on data from five CSA slices [15].

#### Countermovement Jump Test

Each subject performed 5 maximal CMJs with a 20-s rest between each jump. The warm-up protocol included 5 minutes of jogging at a self-selected easy pace, followed by 2 sets of 10 bodyweight SQs, and 2 sets of submaximal CMJs (5 and 3 repetitions, respectively. Flight time values measured by an infrared timing system (OptojumpNext; Microgate, Bolzano, Italy) determined the jump height. For analysis, the highest and the lowest CMJ height values were discarded, and the resulting average of 3 CMJs was kept for the study [16].

#### Maximal Voluntary Isometric Contraction Test

This test consisted of performing a maximal isometric SQ at 90º against an insurmountable resistance to examine the maximal isometric force (MIF) and different force-time parameters. The test was performed on an 80 × 80 cm dynamometric platform (FP-500; Ergotech, Murcia, Spain) and a Smith machine with adjustable height movable supports (MultiPower Fitness Line, Peroga, Murcia, Spain). The subjects were trained to maintain a constant minimum pressure until the researcher ordered the next verbal instruction: “push against the bar as hard and as fast as possible” during two 5-s trials separated by 1 minute. The warm-up test consisted of 2 attempts at 50% and 75% maximum effort with 30 s of rest between them. The software (T-Force System, Ergotech, Murcia, Spain) was used to store the raw data related to the force-time (4^th^ order low-pass Butterworth filter without phase shift with a cut-off frequency of 200 Hz). During each attempt, the following parameters were analyzed: a) maximal isometric force (MIF), b) maximal rate of force development (RFDmax) in time intervals of 20 ms, c) the average of the force-time curve from the onset of force in different time intervals (RFD_0–50_, RFD_0–100_, RFD_0–150_; RFD_0–200_, and RFD_0–400_, respectively). For the analysis, the mean value of the two attempts was used. When the signal rose above 2 standard deviations (SDs) from the baseline signal, the onset of the force signal was established.

#### Progressive Loading Test

This test was conducted to calculate the individual load–velocity relationship (absolute and relative) and the 1RM load in the SQ exercise. Subjects were required to perform a correct execution as described below: a) subjects had to start from the upright position on a Smith machine (Multipower Fitness Line, Peroga, Murcia, Spain) with the knees and hips fully extended, parallel feet and approximately shoulder width apart; b) the barbell had to be across the back at the level of the acromion; c) each subject had to descend in a continuous motion as low as possible (~35~40º knee flexion, with 180º being full extension), and then immediately ascend back to the upright position. The concentric phase was always performed at maximal velocity. Subjects were instructed not to jump or detach the bar from their neck during the concentric phase. The range of movement and the mean propulsive velocity (MPV) of every repetition were recorded at 1000 Hz using a linear velocity transducer (T-Force System; Ergotech, Murcia, Spain), whose reliability has been previously reported [1]. The subjects performed 6 SQ repetitions with a 20-kg load during the warm-up. The test began with 20 kg, gradually increasing by 10 kg until the MPV fell below 0.5 m · s^−1^, indicating a load exceeding 90% 1RM [17]. However, close to the target velocity (MPV < 0.6 m · s^−1^), smaller increments (2.5 or 5 kg) were applied to ensure better adjustments. A total of 8.2 ± 1.7 increasing loads were used for every subject. The number of repetitions performed was as follows: 3 repetitions for light loads (≥ 1.00 m · s^−1^), 2 for moderate loads (1.00–0.80 m · s^−1^), and 1 for the heaviest loads (≤ 0.80 m · s^−1^). Strong verbal encouragement was provided to motivate participants to give a maximal effort. Inter-set recoveries were 3 minutes. For further analyses, the highest MPV with each load was considered. The identical absolute load progression was carried out at the post-training test. The 1RM was determined from the individual linear load-velocity relationship as the load that could be lifted at 0.32 m · s^−1^ [17]. In addition to the 1RM load and the individual load–velocity relationship, the following variables were also calculated: a) average MPV attained against all absolute loads common to pre- and Post-training (AV), b) average MPV attained against absolute loads that were moved faster than 1 m · s^−1^ at Pre-training (AV > 1), and c) average MPV attained against absolute loads that were moved slower than 1 m · s^−1^ at Pre-training (AV < 1). These parameters show potential adaptations achieved in different areas of the load-velocity relationship. The propulsive phase corresponds to the portion of the concentric action during which the measured barbell acceleration is greater than the acceleration due to gravity (-9.81 m · s^−2^) [18].

#### Fatigue test

Five minutes after the progressive loading test, participants completed a fatigue test by performing the maximum number of repetitions (MNR) at 70% 1RM until the MPV dropped below 0.50 m · s^−1^, using the same load for Pre- and Post-training tests. The technique execution and setting were similar to those previously described. For further analysis, the following variables were calculated: a) MNR and b) average MPV attained against the same number of repetitions to Pre-training and Post-training (AV-MNR) (i.e., if one participant performed 8 MNR in Pre-training and 12 MNR in Post-training, the average MPV over the first 8 repetitions in both tests was calculated).

#### EMG signal acquisition

EMG signal was recorded during all SQ tests using a bipolar surface electromyographic sensor (Trigno™ wireless EMG system, Delsys Inc., Natick, MA), with an interelectrode distance of 10 mm, common mode rejection ratio > 80 dB, and bandwidth filter between 20 and 450 Hz ± 10%. EMG electrodes were placed over the distal part of the vastus lateralis muscle of the right leg, following the SENIAM recommendations for EMG sensor locations of individual muscles [19]. The locations were drawn with a permanent marker to replicate and register positions onto transparent acetate using different anatomic landmarks [19]. Furthermore, to improve EMG recordings, the subjects’ skin was prepared by shaving and cleaning the registration area with alcohol, and the electrodes were held with an elastic mesh to reduce movement. The baseline noise was 5 mV peak to peak, and the sampling rate was 2000 Hz. The raw data from the EMG were stored in digital format using EMG Works Acquisition software (Delsys, Inc.). The highest root mean square (RMS) and the median frequency (MDF) were calculated during each repetition using a moving window of 100 ms with an overlap of 99 ms. Since EMG was measured in all SQ tests, the following variables were obtained: RMS_iso_ (RMS value recorded during the MVIC test); RMS_AV_ (RMS attained against all absolute loads common to Pre- and Post-training); RMS_AV < 1_ (RMS attained against absolute loads that were moved slower than 1 m · s^−1^ at Pre-training); RMS_AV > 1_ (RMS attained against absolute loads that were moved faster than 1 m · s^−1^ at Pre-training); RMS_MNR_ (RMS recorded during the MNR completed at pre-training); MDFiso (MDF values during the MVIC test); MDF_AV_ (MDF during AV); MDF_AV < 1_ (MDF recorded during AV < 1); MDF_AV > 1_ (MDF recorded during AV > 1); and MDF_MNR_ (MDF during MNR).

### Resistance Training Program

The descriptive characteristics of the RT program are presented in Table 1. The three groups trained the SQ exercise twice weekly (48–72 hours apart) for 8 weeks using the same relative intensity, increasing from 70% to 85% 1RM over the 8 weeks. Subjects were trained following the same technique and settings described in the “Progressive Loading Test” section. The groups differed in the accumulated volume for each session: HIG performed 24 (weeks 1–2), 21 (weeks 3–4), 18 (weeks 5–6), and 15 (weeks 7–8) repetitions at 70%, 75%, 80%, and 85% 1RM, respectively; MOD performed 12, 10, 8, and 6 repetitions at 70%, 75%, 80%, and 85% 1RM, respectively; and LOW performed only 3 repetitions per session regardless of the intensity used. Each session consisted of a single set with 10-second rest periods between repetitions to minimize fatigue accumulation during the training session. If the MPV difference between the fastest and subsequent repetitions was between 0.04–0.06 m · s^−1^, the inter-repetition rest period was prolonged to 20 seconds. If the difference was between 0.07–0.09 m · s^−1^, an additional 10 seconds of rest was added between repetitions, and so on. Relative loads were determined from the individual load-velocity relationship obtained from the Progressive Loading Test (*R*^2^ = 0.98 ± 0.02). Therefore, the absolute load (kg) was individually adjusted according to the individual velocity (± 0.03 m · s^−1^) associated with the %1RM set for that session. Previous research has shown that 0.03 m · s^−1^ is the smallest detectable change in MPV in SQ using the setting employed in the present study [20]. Before each training session, participants performed a standardized warm-up consisting of: a) 5 minutes of jogging at a self-selected easy pace, b) 2 × 10 repetitions of SQ (no external load), c) 6-6-4-3 repetitions of SQ with 20 kg, 40%, 50%, and 60% 1RM, respectively, d) 2 repetitions with 70% 1RM (only in sessions 5–16); and e) 1 repetition with 80% 1RM (only in sessions 13–16). A 3-minute rest was always taken between warm-up sets. Performance (i.e., MPV) against the 60% 1RM load during the warmup (the heaviest warm-up load employed in all sessions) was used to analyze the evolution of strength performance during the training intervention. At least two experienced expert researchers supervised all training sessions. The following variables were calculated from the training program conducted by each group: Fastest MPV (average MPV of the fastest repetitions performed in each session); Last MPV (average MPV of the last repetitions performed in each session); MPV all reps (average MPV attained during the entire training program); mean VL (average VL attained during the training program); and total rep (total repetitions performed during the training program).

**TABLE 1:** Descriptive characteristics of the 8-week velocity-based squat training program performed by the three experimental groups

Rep × %1RM	Session 1	Session 2	Session 3	Session 4	Session 5	Session 6	Session 7	Session 8
LOW	3 × 70	3 × 70	3 × 70	3 × 70	3 × 75	3 × 75	3 × 75	3 × 75
MOD	12 × 70	12 × 70	12 × 70	12 × 70	10 × 75	10 × 75	10 × 75	10 × 75
HIG	24 × 70	24 × 70	24 × 70	24 × 70	21 × 75	21 × 75	21 × 75	21 × 75

**Rep × %1RM**	**Session 9**	**Session 10**	**Session 11**	**Session 12**	**Session 13**	**Session 14**	**Session 15**	**Session 16**

LOW	3 × 80	3 × 80	3 × 80	3 × 80	3 × 85	3 × 85	3 × 85	3 × 85
MOD	8 × 80	8 × 80	8 × 80	8 × 80	6 × 85	6 × 85	6 × 85	6 × 85
HIG	18 × 80	18 × 80	18 × 80	18 × 80	15 × 85	15 × 85	15 × 85	15 × 85


**Actually Performed**	**Fastest MPV** (m · s^−1^)		**Last MPV** (m · s^−1^)	**MPV all reps** (m · s^−1^)		**Mean VL** (%)	**Total Rep**	

LOW	0.66 ± 0.03		0.61 ± 0.04	0.63 ± 0.06		7.7 ± 2.2	48.0 ± 0.0	
MOD	0.65 ± 0.03		0.59 ± 0.03	0.59 ± 0.03		10.3 ± 3.5	144.0 ± 0.0^L^	
HIG	0.66 ± 0.05		0.57 ± 0.06	0.59 ± 0.05^L^		14.1 ± 6.9 ^L^	311.8 ± 0.6^L/M^	

The data are presented as mean ± SD, N = 36. LOW: Group that trained with low volume (n = 12); MOD: Group that trained with moderate volume (n = 12); HIG: Group that trained with high volume (n = 12); MPV: Mean propulsive velocity; Fastest MPV: Mean MPV of the fastest repetitions measured in each session (this value represents the average intensity, %1RM, achieved during the training program); Last MPV: Mean MPV of the last repetitions in each session; Mean MPV: Mean MPV achieved throughout the entire training program; mean VL: Average velocity loss attained each set; Total rep: Total number of repetitions performed during the training program. Statistically significant differences (*p* < 0.05) with the MOD protocol: ^M^ and with the LOW group: ^L^.

### Statistical Analyses

Values are expressed as mean ± SD. Normality and homoscedasticity were tested with the Shapiro-Wilk and Levene tests, respectively. Data were analyzed using a 3 × 2 factorial ANOVA with one betweengroups factor (LOW vs. MOD vs. HIG) and one within-group factor (Pre-training vs. Post-training). Bonferroni adjustments were applied for post hoc comparisons. Statistical significance was set at *p* ≤ 0.05. Additionally, ES values were calculated using Hedges’ g on the pooled SD [21] with a dedicated spreadsheet. The remaining statistical analyses were conducted using SPSS version 25.0 (SPSS Inc., Chicago, IL, USA).

## RESULTS

Compliance reached 100%, and no significant differences were observed between groups before training.

### Muscle hypertrophy

**FIG. 2:**
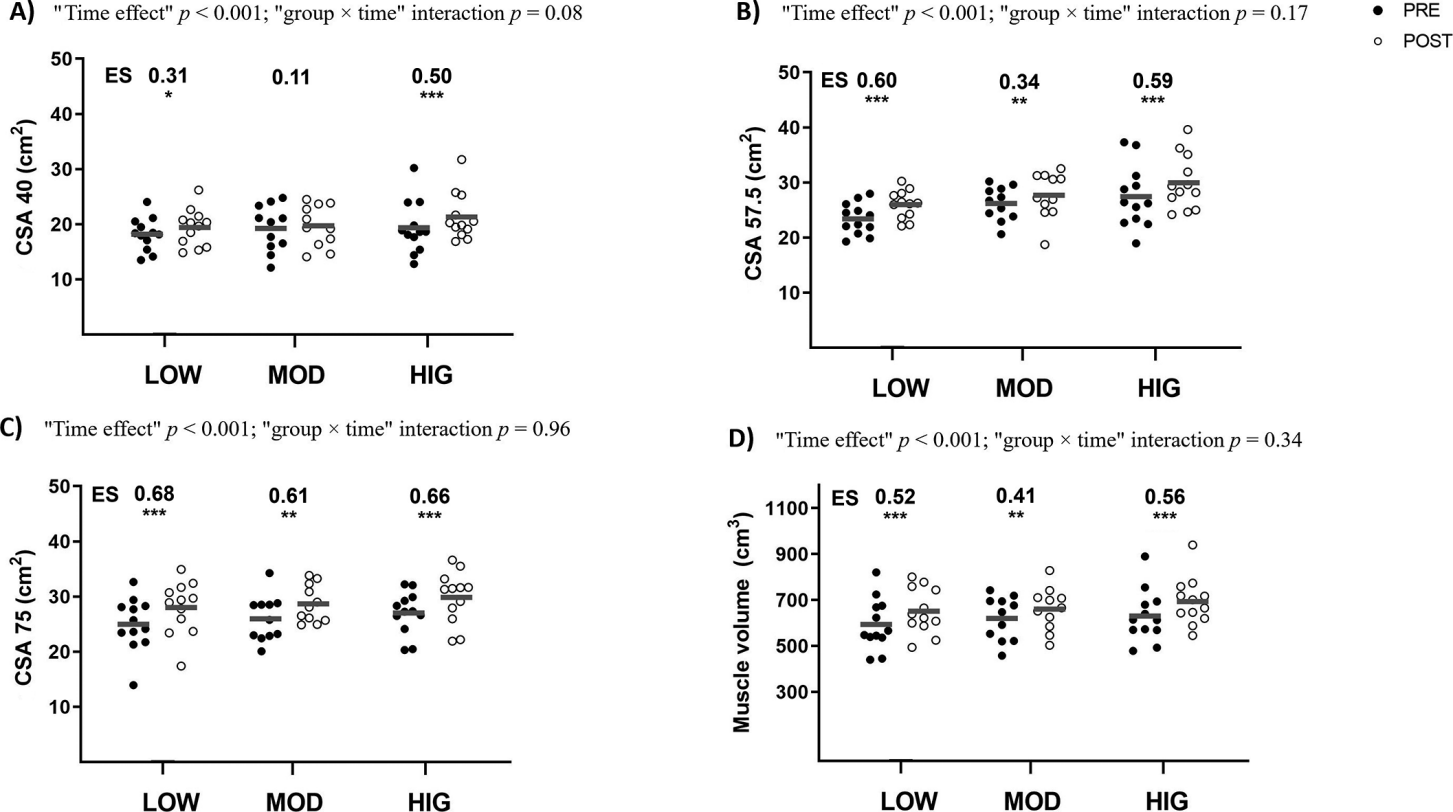
Training-induced adaptations in the anatomical cross-sectional area (CSA) of the vastus lateralis (VLA) muscle. The data are presented as the mean and individual responses, n = 36. LOW: group that trained with low volume; MOD: group that trained with moderate volume; HIG: group that trained with high volume. CSA40: CSA at 40% of the VLA; CSA57.5: CSA at 57.5% of the VLA; CSA75: CSA at 75% of the VLA; Muscle volume: total VLA volume. Statistically significant intra-group differences: *p < 0.05; **p < 0.01; ***p < 0.001. ES, intragroup effect size from Pre- to Post-training.

No significant “group × time” interactions (*p* = 0.08–0.96) were observed, but a significant main “time” effect was found for all variables (Figure 2). All groups achieved significant increases in the different CSAs analyzed (*p* < 0.001–0.05), except MOD for CSA40.

### Maximal Voluntary Isometric Contraction

**FIG. 3:**
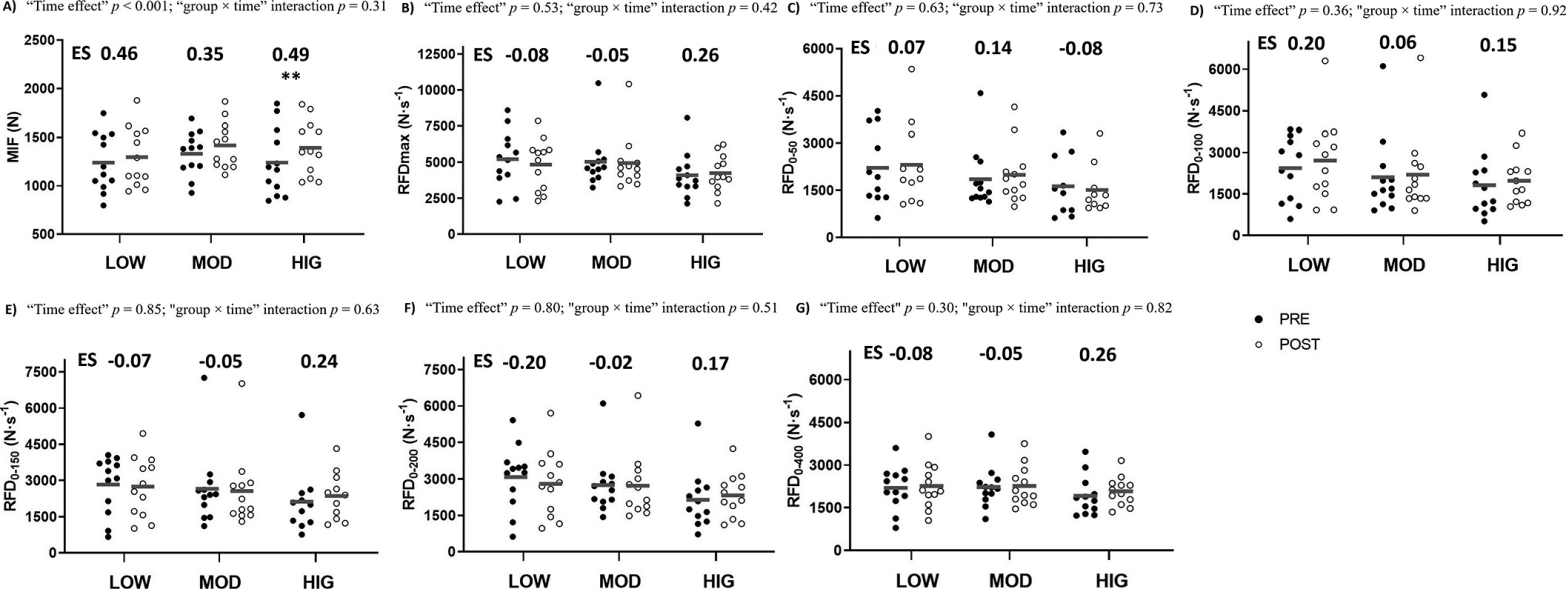
Changes observed from Pre- to Post-training for the three groups in: A) maximal isometric force (MIF); B) maximal rate of force development (RFDmax); C) rate of force development (RFD) from the onset of force production to 50 ms (RFD_0–50_); D) RFD from the onset of force production to 100 ms (RFD_0–100_); E) RFD from the onset of force production to 150 ms (RFD_0–150_); F) RFD from the onset of force production to 200 ms (RFD_0–200_); G) RFD from the onset of force production to 400 ms (RFD_0–400_). Data represent the mean and individual responses, n = 36. LOW: group trained with low volume (n = 12); MOD: group trained with moderate volume (n = 12); HIG: group trained with high volume (n = 12). Significant within-group differences: **p < 0.01. ES: within-group effect size.

No significant “group × time” interactions were observed for the mechanical variables from the MVIC test (Figure 3). No significant “time” effects were observed for these parameters, except for MIF (*p* < 0.001). Only the HIG group showed significant improvements in MIF (*p* < 0.01), with no significant changes in RFD variables.

### Individual load–velocity relationship, resistance to fatigue, and vertical jump

**FIG. 4:**
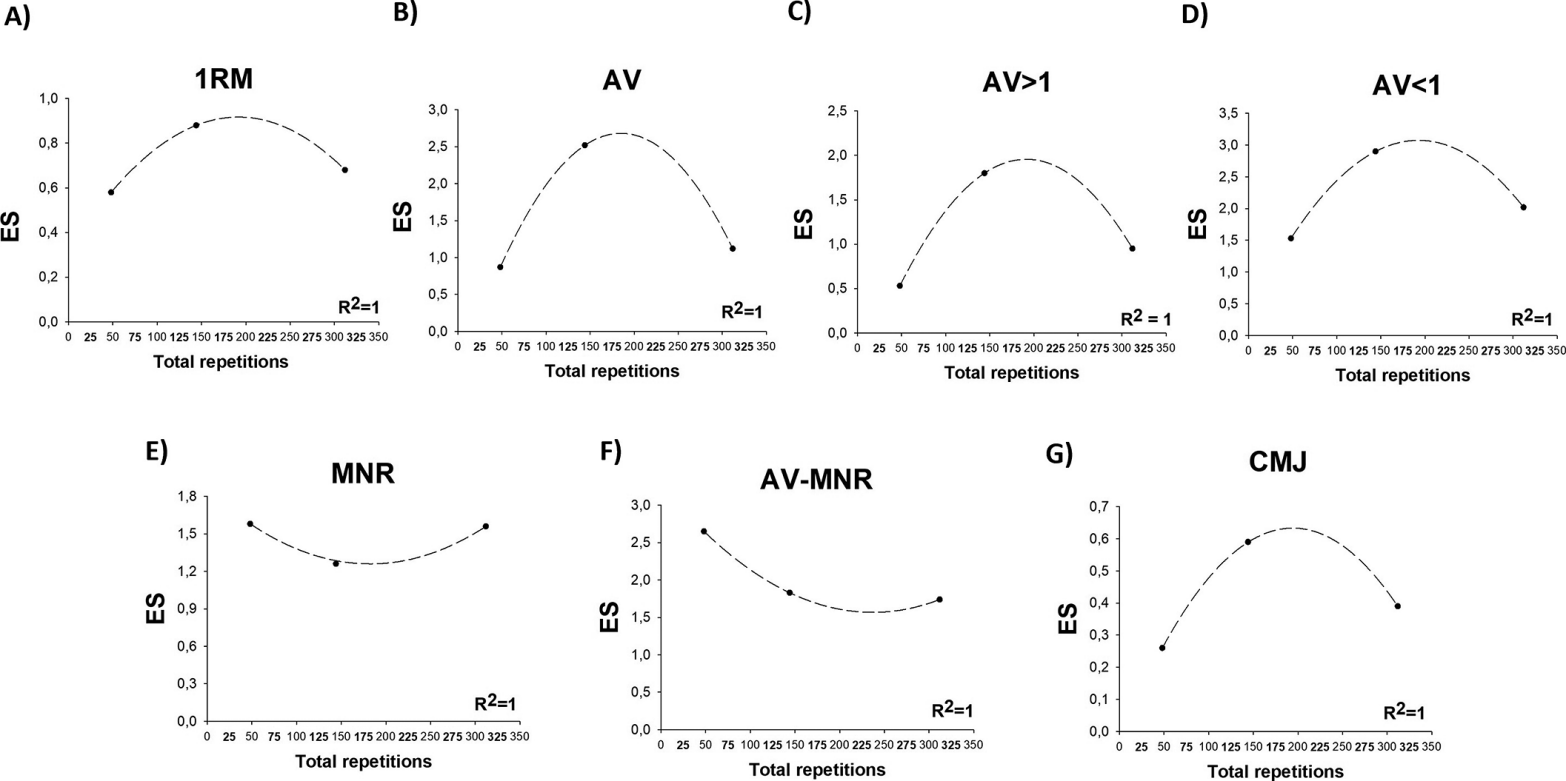
Relationship between full-squat training volume and within-group effect size (ES) from pre- to post-training. A) 1RM, 1-repetition maximum; B) AV, average mean propulsive velocity (MPV) attained against all absolute loads common to pre- and post-training; C) AV ≥ 1, average MPV attained against absolute loads lifted faster than 1.00 m · s^−1^ at pre-training; D) AV < 1, average MPV attained against absolute loads that lifted slower than 1.00 m · s^−1^ at pre-training; E) MNR, maximum number of repetitions in the fatigue test; F) AV_MNR_, average MPV attained against the MNR at pre-training in the fatigue test; G) CMJ, countermovement jump.

**FIG. 5:**
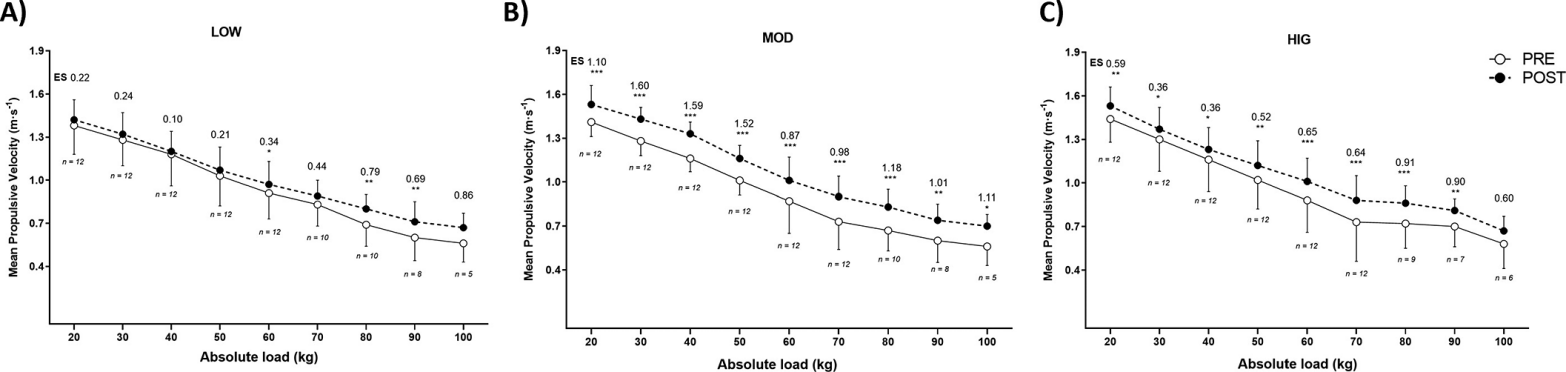
Changes from Pre- to Post-training for the three groups in the absolute load–velocity relationships. Data are mean ± SD, n = 36. LOW: group trained with low volume (n = 12); MOD: group trained with moderate volume (n = 12); HIG: group trained with high volume (n = 12). ES: within-group effect size. Statistically significant differences within group: *p < 0.05; **p < 0.01; ***p < 0.001. The sample size against 70 and 100 kg was lower than previous loads because the participants did not progress to that load during the initial progressive loading test.

Significant “time” effects were detected for all these parameters (*p* < 0.001). Significant “group × time” interactions were found for AV and AV > 1 (*p* < 0.05), with MOD achieving the greatest gains. All groups significantly improved in all variables, except LOW in AV > 1. MOD showed a significantly higher AV > 1 than LOW in the post-training assessment. Likewise, MOD achieved significantly higher CMJ height than HIG at post-training (Table 2). Figure 4 shows the relationship between total volume and performance gains in the different variables analyzed. Figure 5 depicts the changes in the MPV attained against the different absolute loads assessed before and after the training program.

**TABLE 2:** Changes in selected performance variables from Pre- to Post-training for each group.

	LOW			MOD			HIG			p-value	

	Pre	Post	ES	Pre	Post	ES	Pre	Post	ES	time effect	group × time
**1RM** (kg)	101.6 ± 20.9	115.5 ± 24.9^***^	0.58	102.5 ± 18.0	118.9 ± 17.9^***^	0.88	103.7 ± 22.9	117.4 ± 15.6^***^	0.68	< 0.001	0.75

**AV** (m · s^−1^)	0.95 ± 0.10	1.04 ± 0.10^***^	0.87	0.94 ± 0.06	1.11 ± 0.07^***^	2.52	0.94 ± 0.13	1.07 ± 0.09^***^	1.12	< 0.001	0.02

**AV < 1** (m · s^−1^)	0.71 ± 0.04	0.82 ± 0.09^***^	1.53	0.68 ± 0.06	0.86 ± 0.06^***^	2.90	0.71 ± 0.05	0.85 ± 0.08^***^	2.02	< 0.001	0.18

**AV > 1** (m · s^−1^)	1.23 ± 0.10	1.28 ± 0.08	0.53	1.24 ± 0.04	1.38 ± 0.09^***L^	1.80	1.27 ± 0.09	1.35 ± 0.07^**^	0.95	< 0.001	0.02

**MNR** (rep)	9.8 ± 3.8	20.5 ± 8.9^***^	1.56	12.9 ± 4.5	22.6 ± 10.1^***^	1.26	9.1 ± 2.4	20.5 ± 9.5^***^	1.58	< 0.001	0.89

**AV _MNR_** (m · s^−1^)	0.61 ± 0.04	0.75 ± 0.06^***^	2.65	0.61 ± 0.05	0.76 ± 0.10^***^	1.83	0.61 ± 0.04	0.76 ± 0.11^***^	1.74	< 0.001	0.94

**CMJ** (cm)	36.5 ± 7.9	38.6 ± 7.7^**^	0.26	39.1 ± 5.9	42.9 ± 5.8^***H^	0.63	34.2 ± 4.9	36.2 ± 4.9^*^	0.39	< 0.001	0.24

The data are presented as mean ± SD, N = 36. LOW: group that trained with low volume (n = 12); MOD: group that trained with moderate volume (n = 11); HIG: group that trained with high volume (n = 12). 1RM: one-repetition maximum; AV: average mean propulsive velocity (MPV) attained against all absolute loads common to pre-training and post-training; AV < 1: average MPV achieved across absolute loads moved slower than 1.00 m · s^−1^ in Pre-training; AV > 1: average MPV achieved across absolute loads moved faster than 1.00 m · s^−1^ in Pre-training; MNR: maximum number of repetitions in the fatigue test; AV_MNR_: average MPV attained against the MNR completed in Pre-training. CMJ: countermovement jump; ES: intra-group effect size. Significant intra-group differences from Pre- to Post-training:

* *p* ≤ 0.05,

** *p* ≤ 0.01,

*** *p* ≤ 0.001. Statistically significant differences (*p* < 0.05) with the LOW group: ^L^ and with the HIG group: ^H^.

### EMG-derived outcomes

Significant “group × time” interactions were observed for all RMS variables analyzed, except for RMS_AV > 1_, with HIG achieving the greatest increases (Table 3). Only the HIG group showed a significant increase in RMS_ISO_ (*p* < 0.01). HIG and MOD significantly increased RMS_AV_ and RMS_AV < 1_, while no significant changes were recorded for LOW. MOD and HIG significantly increased RMS_MNR,_ but LOW showed significant decreases. No significant “group × time” interactions were detected for the MDF parameters (Table 3).

**TABLE 3:** Changes in selected EMG parameters from Pre- to Post-training for each group

	LOW			MOD			HIG			p-value	

	Pre	Post	ES	Pre	Post	ES	Pre	Post	ES	time effect	group × time
**RMS _ISO_** (mV)	0.25 ± 0.13	0.24 ± 0.12	−0.08	0.29 ± 0.17	0.30 ± 0.19	0.05	0.17 ± 0.09	0.27 ± 0.19**	0.65	0.11	0.03

**RMS _AV_** (mV)	0.34 ± 0.23	0.31 ± 0.22	−0.13	0.29 ± 0.13	0.36 ± 0.13*	0.52	0.17 ± 0.08	0.27 ± 0.13*	0.88	0.02	0.03

**RMS _AV < 1_** (mV)	0.34 ± 0.23	0.31 ± 0.23	−0.13	0.30 ± 0.13	0.39 ± 0.14*	0.64	0.18 ± 0.08	0.28 ± 0.13*	0.88	0.02	0.01

**RMS _AV > 1_** (mV)	0.33 ± 0.23	0.31 ± 0.22	−0.09	0.29 ± 0.13	0.33 ± 0.12	0.31	0.16 ± 0.08	0.25 ± 0.12*	0.84	0.05	0.09

**RMS _MNR_** (mV)	0.33 ± 0.20	0.22 ± 0.10*	−0.66	0.29 ± 0.11	0.38 ± 0.16*	0.56	0.20 ± 0.11	0.30 ± 0.17*	0.86	0.30	0.001

**MDF _ISO_** (Hz)	83.0 ± 17.2	89.9 ± 28.2	0.28	104.1 ± 34.9	104.6 ± 31.4	0.01	80.1 ± 9.7	82.8 ± 23.0	0.09	0.42	0.82

**MDF _AV_** (Hz)	77.9 ± 13.6	89.0 ± 26.0	0.51	91.8 ± 28.3	93.9 ± 30.1	0.07	71.2 ± 6.6	73.6 ± 11.9	0.23	0.13	0.47

**MDF _AV < 1_** (Hz)	79.1 ± 14.5	92.3 ± 27.4	0.58	95.3 ± 29.0	96.7 ± 30.3	0.05	72.3 ± 8.0	77.8 ± 14.0	0.46	0.11	0.46

**MDF AV > 1 iso** (Hz)	75.0 ± 16.8	84.2 ± 26.8	0.40	87.2 ± 28.3	90.4 ± 30.0	0.11	69.6 ± 7.4	69.3 ± 11.8	−0.03	0.23	0.51

**MDF _MNR_** (Hz)	76.9 ± 16.9	85.5 ± 17.4	0.48	92.0 ± 24.9	95.6 ± 28.1	0.13	74.9 ± 9.8	85.0 ± 18.8	0.64	0.02	0.53

The data are presented as mean ± SD, N = 36. LOW: group that trained with low volume (n = 12); MOD: group that trained with moderate volume (n = 12); HIG: group that trained with high volume (n = 12). RMS_iso_: maximum root mean square (RMS) value recorded during the maximal voluntary isometric contraction (MVIC). RMS_AV_: RMS recorded during common loads at pre- and posttraining (AV). RMS_AV < 1_: RMS recorded during common loads at pre- and post-training that moved at less than 1.00 m · s^−1^; RMS_AV_ > 1: RMS recorded during common loads at pre- and post-training that moved at more than 1.00 m · s^−1^. RMS_MNR_: RMS recorded during the fatigue test (MNR). MDF_iso_: median frequency (MDF) values during MVIC. MDF_AV_: MDF during AV. MDF_AV <_ 1: MDF recorded during common loads at pre- and post-training that moved at less than 1.00 m · s^−1^; MDF_AV > 1_: MDF recorded during common loads at pre- and post-training that moved at more than 1.00 m · s^−1^; MDF_MNR_: MDF during MNR. Significant intragroup differences from Pre- to Post-training:

* *p* ≤ 0.05,

* *p* ≤ 0.01.

### Training program

**FIG. 6:**
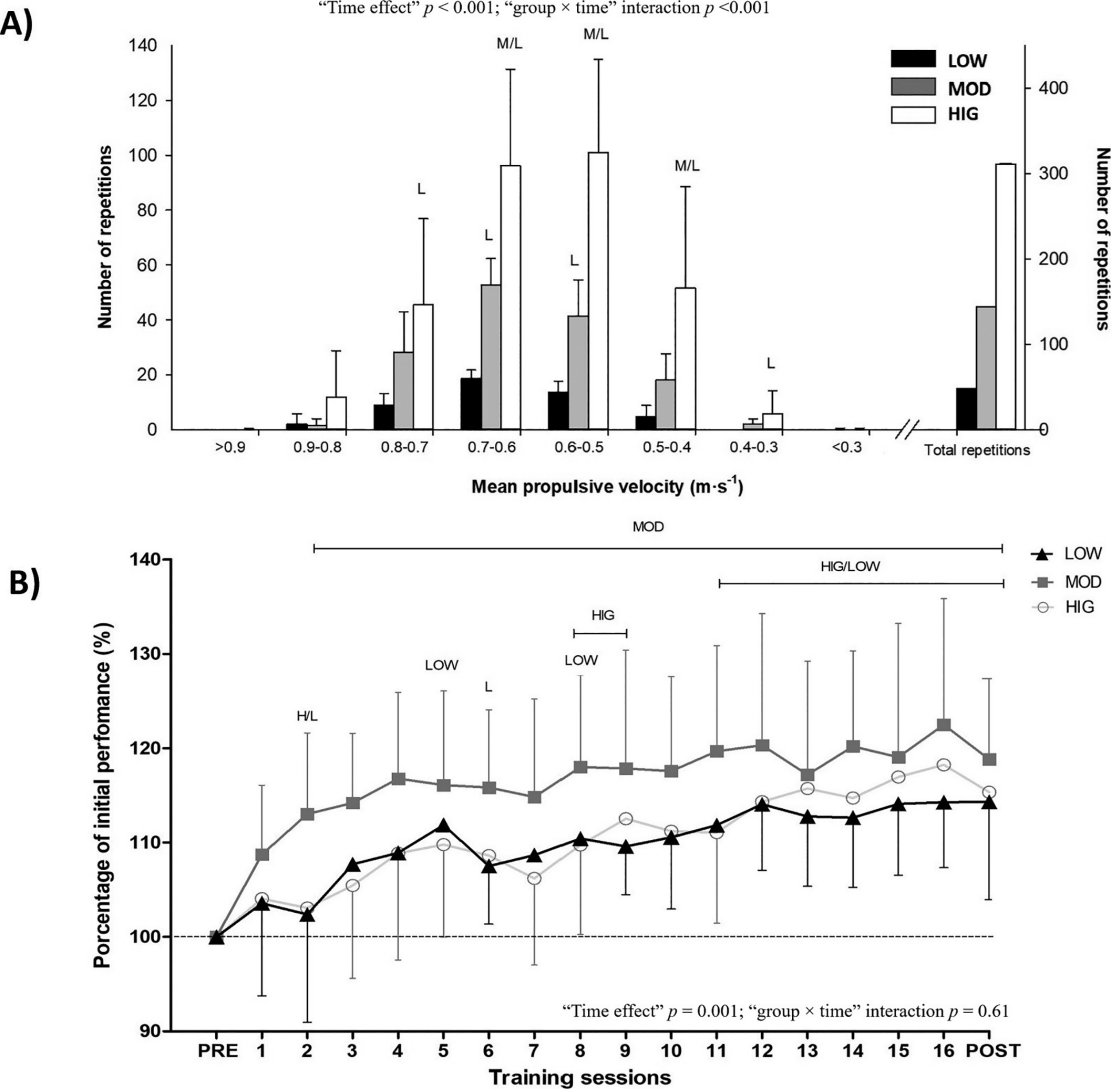
A) Number of repetitions performed in each velocity range and total number of repetitions completed by each training group; B) Evolution of the full-squat performance in every training session expressed as a percentage of the pre-training level for each experimental group. HIG, MOD, and LOW indicate the session at which the respective group achieved significant improvements (*p* < 0.05) compared to their pre-training values. Statistically significant differences (*p* < 0.05) are indicated as follows: ^L^ for comparisons with the LOW group, ^M^ for comparisons with the MOD group, and ^H^ for comparisons with the HIG group.

The average of the fastest repetitions, representing the %1RM lifted, was very similar for all groups (Table 1). The HIG group achieved lower MPV all reps and higher VL than the LOW group throughout the training program. The total repetitions performed at different velocity ranges are shown in Figure 6A. Figure 6B displays the evolution of daily performance in each training session for all groups.

## DISCUSSION

To the best of our knowledge, this is the first study to evaluate the effects of lower-body RT volume while controlling for the impact of fatigue on strength gains, neuromuscular adaptations, and muscle hypertrophy. To mitigate the fatigue accumulation associated with higher volumes, a short rest period was introduced after each repetition, individualized based on the performance achieved in the previous repetition. Despite the considerable differences in the training volume accumulated by each group (LOW: 48 repetitions; MOD: 144 repetitions; HIG: 312 repetitions), no significant differences were observed in many of the analyzed variables. Interestingly, when differences were detected, moderate training volume consistently provided the greatest improvements. These findings suggest that while achieving a certain training volume is necessary for eliciting neuromuscular adaptations, exceeding this threshold does not enhance strength gains consequent to RT with inter-repetition rest periods, and may even attenuate the benefits achieved with lower volumes.

Regarding muscle hypertrophy, all groups achieved similar positive adaptations in the CSA of the vastus lateralis muscle. A longheld view supported by some meta-analyses is that RT programs can promote muscle growth when performed close to muscle failure [22]. A previous study on different SQ training programs found that only VL20 and VL40 protocols induced significant gains in vastus lateralis CSA (~5–7%), while VL0 and VL10 protocols resulted in non-significant ~2% increases [3]. Another study found that both VL20 and VL40 increased quadriceps CSA, with VL40 training resulting in greater muscle hypertrophy [6]. These findings suggest that in continuous repetition RT, increasing the training volume, and fatigue, up to a certain point led to greater gains in muscle CSA. In contrast, we here report similar vastus lateralis hypertrophy (~7–10% in both CSA and muscle volume) between the LOW, MOD, and HIG groups, despite the differences in accumulated training volume. Furthermore, the ~7–10% gains in muscle volume and CSA for the LOW and MOD groups in this study appear to be greater than the ~2% gains in vastus lateralis CSA in the VL0 and VL10 groups in a previous study [3], in which these groups performed the same type of SQ exercise with almost identical total number of repetitions and load progressions as the LOW and MOD groups, respectively. Taken together, these findings suggest that brief ~10–30-second rest periods between repetitions can increase the efficacy of low-volume and moderate-volume strength training in terms of inducing vastus lateralis muscle hypertrophy. One possible explanation is that such rest-redistribution strategies allow greater maintenance of mechanical tension across repetitions [23]. Enhanced mechanical tension stimulates muscle growth via increased mechanotransduction and protein synthesis [24]. Moreover, the warm-up could have acted as an stimulus contributing to hypertrophic adaptations. Future studies should directly test this hypothesis.

It is well established that protocols involving higher levels of effort within the set (i.e., higher VL) increase mechanical and metabolic stress [1], along with elevating hormonal responses and causing muscle damage [2]. The key factors contributing to hypertrophic adaptations are mechanical stress, muscle damage, and metabolic fatigue [25]. In this study, no group performed slow, fatiguing repetitions close to muscle failure, as rest periods were added between repetitions and extended if fatigue was observed through lifting velocity. Indeed, the three groups performed approximately 85% of their repetitions at velocities between 0.5 and 0.9 m · s^−1^ (LOW: 90%; MOD: 86%; HIG: 82%) with about 15% occurring between 0.3 and 0.5 m · s^−1^ (LOW: 10%; MOD: 14%; HIG: 18%). The similar VL and average MPV across groups suggest that hypertrophic responses depend more on the fatigue level in each set than on training volume. Thus, by isolating volume as an independent variable, we demonstrate for the first time that training volume may play a secondary role in the muscle hypertrophic adaptations resulting from heavy strength training with inter-repetition rest periods.

With reference to strength-related outcomes, all groups significantly enhanced their 1RM strength (LOW: +13.7%, MOD: +16.4%, and HIG: +14.0%). Notably, performing just 3 SQ repetitions at 70–85% 1RM twice per week (LOW group) was sufficient to induce substantial strength gains, despite representing a much lower training dose compared to MOD and HIG groups. A previous review has suggested that the minimal dose for improving 1RM strength consists of a single set of 6 to 12 repetitions to failure with loads of 70–85% 1RM over 8–12 weeks, with a frequency of 2–3 sessions per week [26]. However, our findings indicate that even lower training doses of high loads (≥ 70–85% 1RM) can effectively enhance maximal dynamic strength. Nevertheless, it is important to acknowledge the potential contribution of the standardized warm-up protocol, which included progressive sets at increasing intensities (6, 6, 4, and 3 repetitions with 20 kg, 40%, 50%, and 60% 1RM, respectively) along with 1–2 additional repetitions at 70% and 80% 1RM in later sessions. This warm-up may have served as an additional training stimulus, particularly benefiting strength gains in the LOW group.

In contrast, the HIG group was the only one to achieve improvements in MIF (~20%). Consistently, a previous study comparing four VL thresholds reported that MIF improvements increased as VL thresholds rose (VL0: 8.0%, VL10: 11.1%, VL20: 11.4%, VL40: 14.3%) [3]. This finding suggests that for MIF, where the time required to reach peak force is not a critical factor and movement velocity is zero, accumulating a higher number of repetitions may provide additional benefits. However, there were no clear effects of training volume on the different RFD variables.

Regarding load-velocity performance at submaximal loads, moderate volume training produced the greatest improvements. Consequently, increasing the training volume may attenuate the velocity gains induced by moderate training volumes, while a low volume may be insufficient to maximize performance gains under these loads. Pareja-Blanco et al. [3] reported that only the VL10 and VL20 groups showed improvements in high-velocity actions (i.e., AV > 1), while the VL0 and VL40 groups did not exhibit significant increases. In contrast, the HIG group in this study increased in high-velocity actions across several loads. Since the HIG group performed as many total repetitions as the VL40 group in the study of Pareja-Blanco et al., [3] this suggests that even relatively high volumes can lead to high-velocity gains as long as fatigue is minimized during the sets.

Nonetheless, our findings suggest that moderate training volumes may provide a more optimal stimulus for gains in force-velocity performance tests also when heavy strength training is performed with inter-repetition rest periods. This suggestion is supported by the consistently greater ES values obtained by the MOD group at all loads, especially in high-velocity performance (≥ 1.00 m · s^−1^), and also by the MOD group surpassing the HIG group in CMJ height post-training (*p* < 0.05). These findings suggest that performing additional repetitions, even with minimal fatigue, may not be an effective strategy for maximizing jump performance gains. A further advantage of lower volume training protocols is their faster recovery rates, whereas higher training volumes require longer recovery periods, sometimes exceeding 48 hours [27]. Additionally, previous research has reported a decline in CMJ performance when high training volumes are conducted to failure compared to non-failure training [28].

Traditionally, it has been widely accepted that a high number of repetitions per set is necessary to maximize muscular endurance gains [29, 30]. However, despite significant differences in training volume, all groups obtained notable improvements in muscular endurance performance (MNR, ES; LOW: 1.56; MOD: 1.26; HIG: 1.58). These findings suggest that muscular endurance gains in the SQ exercise after resistance training with inter-repetition rest periods may not be directly dependent on training volume. Previous research reported greater endurance improvements in bench press at 75% 1RM when training to failure compared to non-failure training, yet no significant differences were observed in SQ [31]. However, it is important to note that Izquierdo et al. [5] compared matched-volume protocols with differing fatigue levels (failure vs. non-failure), whereas the present study aimed to isolate the effects of training volume by minimizing fatigue accumulation. This fundamental difference may account for the discrepancies in findings and highlights the need for further research to disentangle the independent effects of volume and fatigue on muscular endurance adaptations.

To explore the neuromuscular mechanisms underlying performance changes in the RT program, EMG signals were analyzed during strength tests. Significant increases in RMS were observed during the MVIC test for the HIG group, as well as during the progressive loading and fatigue tests for the MOD group (3 out of 5 tests) and the HIG group (5 out of 5 tests). In contrast, the LOW group did not show any increases in EMG RMS and even a decrease in the NMR test. The rise in EMG RMS observed in HIG but not in LOW suggests that a greater number of repetitions, when performed with preserved movement quality, is required to elicit neuromuscular adaptations. The use of short inter-repetition rests likely allowed subjects to maintain high-quality repetitions, enabling cumulative motor unit (MU) recruitment and high firing rates in HIG. Conversely, the minimal volume in LOW was insufficient to trigger such responses. Previous studies have similarly reported increases in RMS during 1RM loading [32, 33] and MVIC [34, 35]. In addition to MU recruitment and firing rates, RMS changes may also be attributed to alterations in muscle fiber type composition, muscle size, muscle fiber excitability, and calcium sensitivity, all of which contribute to neuromuscular adaptations [36–41]. By contrast, the MDF data were inconclusive, showing no significant changes for any group. Since MDF increases in proportion to MU recruitment [42], the lack of significant changes suggests no major improvements in MU recruitment in response to any of the programs. However, since EMG RMS also increases with increased MU firing rates [40, 41], which are not necessarily reflected in MDF measurements it seems possible that higher MU firing rates could also have contributed to the increased EMG RMS values observed for the MOD and HIG groups in some of the tests. Consequently, the observed performance improvements may be explained by one or more of these neuromuscular adaptations. Regardless, more extensive investigations are needed to clarify which of these adaptations are most responsible for driving the increases in strength and force-velocity performance observed in this study.

Several limitations should be considered when interpreting our findings. Firstly, energy and protein intake were not controlled during the study, which could affect the adaptations induced by the RT program. Secondly, the RFD findings should be interpreted cautiously, as the reliability of the RFD measurements was limited (CV from 13% to 19%). Thirdly, muscle size changes were investigated only in the vastus lateralis, and we cannot rule out that other muscles in the quadriceps and/or in other muscle groups may have responded differently to the training programs. Finally, several fitness tests were conducted on the same day (CMJ, MVIC, progressive loading test, and fatigue test), which may have caused fatigue in participants. While all groups were likely affected similarly, this could have impacted the accuracy of the tests in measuring the intended outcomes. Future studies should investigate the chronic effects of RT with stable MPV during a set compared to traditional sets (characterized by a decrease in MPV within the set).

## CONCLUSIONS

High-volume SQ training does not yield additional strength gains and may even attenuate the strength-related adaptations stimulated by earlier repetitions. However, a low volume may not be sufficient to maximize training adaptations. Overall, there appears to be an inverted U-shaped relationship between training volume and strength gains. Our findings also indicate that when fatigue levels are matched, by using individualized inter-repetition rest, higher training volumes do not result in greater muscle hypertrophy compared to lower volumes. However, a certain training volume appears to be necessary to evoke marked neuromuscular adaptations.

Taken together, it could be concluded that the effects of training volume and fatigue depend on the variable analyzed. Strength gains rely on the quality of the repetitions, avoiding excessive fatigue. However, the opposite seems to be true for hypertrophy, where fatigue appears to be required to maximize muscle mass gains. However, it seems that to maximize neuromuscular adaptations, a high volume of high-quality repetitions is required.

Athletes and coaches should avoid excessively high volumes that could hinder progress and instead aim for a moderate volume that is sufficient to stimulate neuromuscular adaptations and maximize strength gains. This involves carefully monitoring training loads to ensure that workouts provide enough stimulus without leading to fatigue that could compromise performance. Additionally, velocity monitoring allows coaches to assess individual responses to training to tailor programs accordingly, ensuring that the training volume and fatigue generated align with the athlete’s goals, experience level, and recovery capacity for optimal results.

## References

[1] Sánchez-Medina L, González-Badillo JJ. Velocity loss as an indicator of neuromuscular fatigue during resistance training. Med Sci Sports Exerc. 2011; 43(9), 1725–1734. 21311352 10.1249/MSS.0b013e318213f880

[2] Moran-Navarro R, Pérez CE, Mora-Rodríguez R, et al. Time course of recovery following resistance training leading or not to failure. Eur J Appl Physiol. 2017; 117(12):2387–99. 28965198 10.1007/s00421-017-3725-7

[3] Pareja-Blanco F, Alcázar J, Sánchez-Valdepeñas J, et al. Velocity loss as a critical variable determining the adaptations to strength training. Med Sci Sports Exerc. 2020; 52(8):1752–62. 32049887 10.1249/MSS.0000000000002295

[4] Rodríguez-Rosell D, Yáñez-García JM, Sánchez-Medina L, Mora-Custodio R, González-Badillo JJ. Relationship between velocity loss and repetitions in reserve in the bench press and back squat exercises. J Strength Cond Res. 2020; 34(9):2537–47. 31045753 10.1519/JSC.0000000000002881

[5] Izquierdo M, González-Badillo JJ, Häkkinen K, et al. Effect of loading on unintentional lifting velocity declines during single sets of repetitions to failure during upper and lower extremity muscle actions. Int J Sports Med. 2006; 27(9):718–24. 16944400 10.1055/s-2005-872825

[6] Pareja-Blanco F, Rodríguez-Rosell D, Sánchez-Medina L, et al. Effects of velocity loss during resistance training on athletic performance, strength gains, and muscle adaptations. Scand J Med Sci Sports. 2017; 27(7):724–35. 27038416 10.1111/sms.12678

[7] Rodiles-Guerrero L, Sánchez-Valdepeñas J, Cornejo-Daza PJ, et al. Effects of velocity loss during bench-press training with light relative loads. Int J Sports Physiol Perform. 2024; 1–11. 10.1123/ijspp.2023-0529 39168458

[8] Brigatto FA, Lima LEM, Germano MD, Aoki MS, Braz TV, Lopes CR. High resistance-training volume enhances muscle thickness in resistance-trained men. J Strength Cond Res. 2022; 36(1):22–30. 31868813 10.1519/JSC.0000000000003413

[9] Schoenfeld BJ, Contreras B, Krieger J, et al. Resistance training volume enhances muscle hypertrophy but not strength in trained men. Med Sci Sports Exerc. 2019; 51(1):94–103. 30153194 10.1249/MSS.0000000000001764 PMC6303131

[10] Schoenfeld BJ, Ogborn D, Krieger JW. Dose-response relationship between weekly resistance training volume and increases in muscle mass: a systematic review and meta-analysis. J Sports Sci. 2017; 35(11):1073–82. 27433992 10.1080/02640414.2016.1210197

[11] Ortega-Becerra M, Sánchez-Moreno M, Pareja-Blanco F. Effects of cluster set configuration on mechanical performance and neuromuscular activity. J Strength Cond Res. 2021; 35(2):310–7. 33278270 10.1519/JSC.0000000000003907

[12] Tufano JJ, Conlon JA, Nimphius S, Oliver JM, Kreutzer A, Haff GG. Different cluster sets result in similar metabolic, endocrine, and perceptual responses in trained men. J Strength Cond Res. 2019; 33(2):346–54. 28301435 10.1519/JSC.0000000000001898

[13] Páez-Maldonado JA, Cano C, Cornejo-Daza PJ, et al. Effects of training volume in the bench-press exercise performed with interrepetition rest periods on strength gains and neuromuscular adaptations. Int J Sports Physiol Perform. 2024; 20(1):37–46. 39481368 10.1123/ijspp.2024-0228

[14] Hogrel JY, Barnouin Y, Azzabou N, et al. NMR imaging estimates of muscle volume and intramuscular fat infiltration in the thigh: variations with muscle, gender, and age. Age (Dordr). 2015; 37(3):9798. 26040416 10.1007/s11357-015-9798-5 PMC4456487

[15] Cornejo-Daza PJ, Sánchez-Valdepeñas J, Rodiles-Guerrero L, et al. Vastus lateralis muscle size is differently associated with the different regions of the squat force-velocity and load-velocity relationships, rate of force development, and physical performance in young men. J Strength Cond Res. 2024; 38(3):450–8. 38231131 10.1519/JSC.0000000000004654

[16] Claudino JG, Cronin J, Mezêncio B, et al. The countermovement jump to monitor neuromuscular status: a meta-analysis. J Sci Med Sport. 2017; 20(4):397–402. 27663764 10.1016/j.jsams.2016.08.011

[17] Sánchez-Medina L, Pallarés JG, Pérez CE, Moran-Navarro R, González-Badillo JJ. Estimation of relative load from bar velocity in the full back squat exercise. Sports Med Int Open. 2017; 1(2): E80–8. 30539090 10.1055/s-0043-102933 PMC6226068

[18] Sánchez-Médina L, Pérez CE, González-Badillo JJ. Importance of the propulsive phase in strength assessment. Int J Sports Med. 2010; 31(2):123–9. 20222005 10.1055/s-0029-1242815

[19] Hermens HJ, Freriks B, Disselhorst-Klug C, Rau G. Development of recommendations for SEMG sensors and sensor placement procedures. J Electromyogr Kinesiol. 2000; 10(5):361–74. 11018445 10.1016/s1050-6411(00)00027-4

[20] Courel-Ibáñez J, Martínez-Cava A, Morán-Navarro R, et al. Reproducibility and repeatability of five different technologies for bar velocity measurement in resistance training. Ann Biomed Eng. 2019; 47(7):1523–38. 30980292 10.1007/s10439-019-02265-6

[21] Hedges LV, Olkin O. Estimation of a single effect size: parametric and nonparametric method. In: Statistical Methods for Meta-Analysis. San Diego, CA: Academic Press; 1985. p. 76–108.

[22] Schoenfeld BJ, Pope ZK, Benik FM, et al. Longer interset rest periods enhance muscle strength and hypertrophy in resistance-trained men. J Strength Cond Res. 2016; 30(7):1805–12. 26605807 10.1519/JSC.0000000000001272

[23] Tufano JJ, Conlon JA, Nimphius S, et al. Cluster Sets: Permitting Greater Mechanical Stress Without Decreasing Relative Velocity. Int J Sports Physiol Perform. 2017; 12(4):463–469. 27617387 10.1123/ijspp.2015-0738

[24] Behringer M, Heinrich C, Franz A. Anabolic signals and muscle hypertrophy– significance for strength training in sports medicine. Sports Orthop. Traumatology. 2025; 41(1):9–18.

[25] Schoenfeld BJ. The mechanisms of muscle hypertrophy and their application to resistance training. J Strength Cond Res. 2010; 24(10):2857–72. 20847704 10.1519/JSC.0b013e3181e840f3

[26] Androulakis-Korakakis P, Fisher JP, Steele J. The minimum effective training dose required to increase 1RM strength in resistance-trained men: a systematic review and meta-analysis. Sports Med. 2020; 50(4):751–65. 31797219 10.1007/s40279-019-01236-0

[27] Pareja-Blanco F, Villalba-Fernández A, Cornejo-Daza PJ, Sánchez-Valdepeñas J, González-Badillo JJ. Time course of recovery following resistance exercise with different loading magnitudes and velocity loss in the set. Sports (Basel). 2019; 7(3):59. 30836680 10.3390/sports7030059 PMC6473797

[28] Carroll KM, Bazyler CD. Skeletal muscle fiber adaptations following resistance training using repetition maximums or relative intensity. Sports (Basel). 2019; 7(7):169. 31373325 10.3390/sports7070169 PMC6680702

[29] Bird SP, Tarpenning KM, Marino FE. Designing resistance training programmes to enhance muscular fitness: a review of the acute programme variables. Sports Med. 2005; 35(10):841–51. 16180944 10.2165/00007256-200535100-00002

[30] Kraemer WJ, Ratamess NA. Fundamentals of resistance training: progression and exercise prescription. Med Sci Sports Exerc. 2004; 36(4):674–688. 15064596 10.1249/01.mss.0000121945.36635.61

[31] Izquierdo M, Ibañez J, González--Badillo JJ, et al. Differential effects of strength training leading to failure versus not to failure on hormonal responses, strength, and muscle power gains. J Appl Physiol (1985). 2006; 100(5):1647–56. 16410373 10.1152/japplphysiol.01400.2005

[32] Buckthorpe M, Erskine RM, Fletcher G, Folland JP. Task-specific neural adaptations to isoinertial resistance training. Scand J Med Sci Sports. 2015; 25(5):640–9. 25077768 10.1111/sms.12292

[33] Sampson JA, Groeller H. Is repetition failure critical for the development of muscle hypertrophy and strength? Scand J Med Sci Sports. 2016; 26(4):375–83. 25809472 10.1111/sms.12445

[34] Aagaard P, Simonsen EB, Andersen JL, Magnusson P, Dyhre-Poulsen P. Increased rate of force development and neural drive of human skeletal muscle following resistance training. J Appl Physiol (1985). 2002; 93(4):1318–26. 12235031 10.1152/japplphysiol.00283.2002

[35] Ullrich B, Holzinger S, Soleimani M, Pelzer T, Stening J, Pfeiffer M. Neuromuscular responses to 14 weeks of traditional and daily undulating resistance training. Int J Sports Med. 2015; 36(7):554–62. 25760153 10.1055/s-0034-1398529

[36] Gabriel DA, Kamen G, Frost G. Neural adaptations to resistive exercise: mechanisms and recommendations for training practices. Sports Med. 2006; 36(2):133–49. 16464122 10.2165/00007256-200636020-00004

[37] Semmler JG. Motor unit synchronization and neuromuscular performance. Exerc Sport Sci Rev. 2002; 30(1):8–14. 11800501 10.1097/00003677-200201000-00003

[38] Škarabot J, Balshaw TG, Maeo S, et al. Neural adaptations to long-term resistance training: evidence for the confounding effect of muscle size on the interpretation of surface electromyography. J Appl Physiol (1985) 2021; 131(2), 702–15. 34166110 10.1152/japplphysiol.00094.2021

[39] Pucci AR, Griffin L, Cafarelli E. Maximal motor unit firing rates during isometric resistance training in men. Exp Physiol 2006; 91(1):171–8. 16210447 10.1113/expphysiol.2005.032094

[40] Bigland-Ritchie B, Jones DA, Woods JJ. Excitation frequency and muscle fatigue: electrical responses during human voluntary and stimulated contractions. Exp Neurol. 1979; 64(2):414–27. 428516 10.1016/0014-4886(79)90280-2

[41] Christie A, Greig Inglis J, Kamen G, Gabriel DA. Relationships between surface EMG variables and motor unit firing rates. Eur J Appl Physiol. 2009; 107(2):177–85. 19544067 10.1007/s00421-009-1113-7

[42] Solomonow M, Baten C, Smit J, et al. Electromyogram power spectra frequencies associated with motor unit recruitment strategies. J Appl Physiol (1985). 1990; 68(3):1177–85. 2341343 10.1152/jappl.1990.68.3.1177

